# Supplementary figures and images for: Genome characterization based on the Spike-614 and NS8-84 loci of SARS-CoV-2 reveals two major possible onsets of the COVID-19 pandemic
Source: PLoS One. 2023 Jun 15;18(6):e0279221. doi: 10.1371/journal.pone.0279221 (PMC10270620; doi:10.1371/journal.pone.0279221)

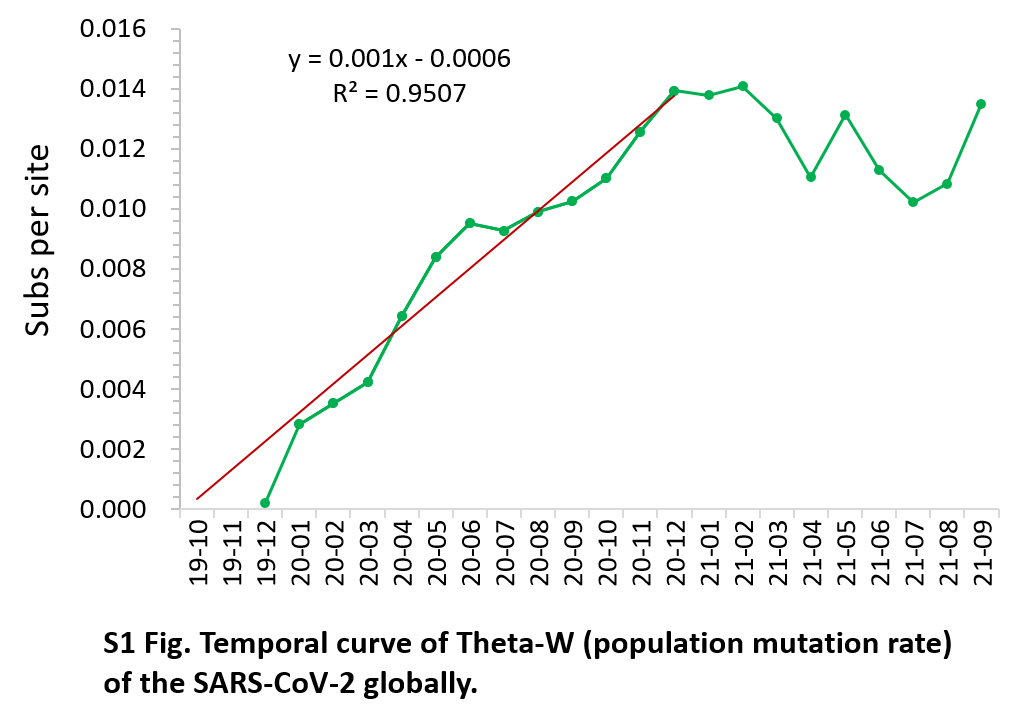

Supplement: S1 Fig — (TIF) [file pone.0279221.s001.tif]

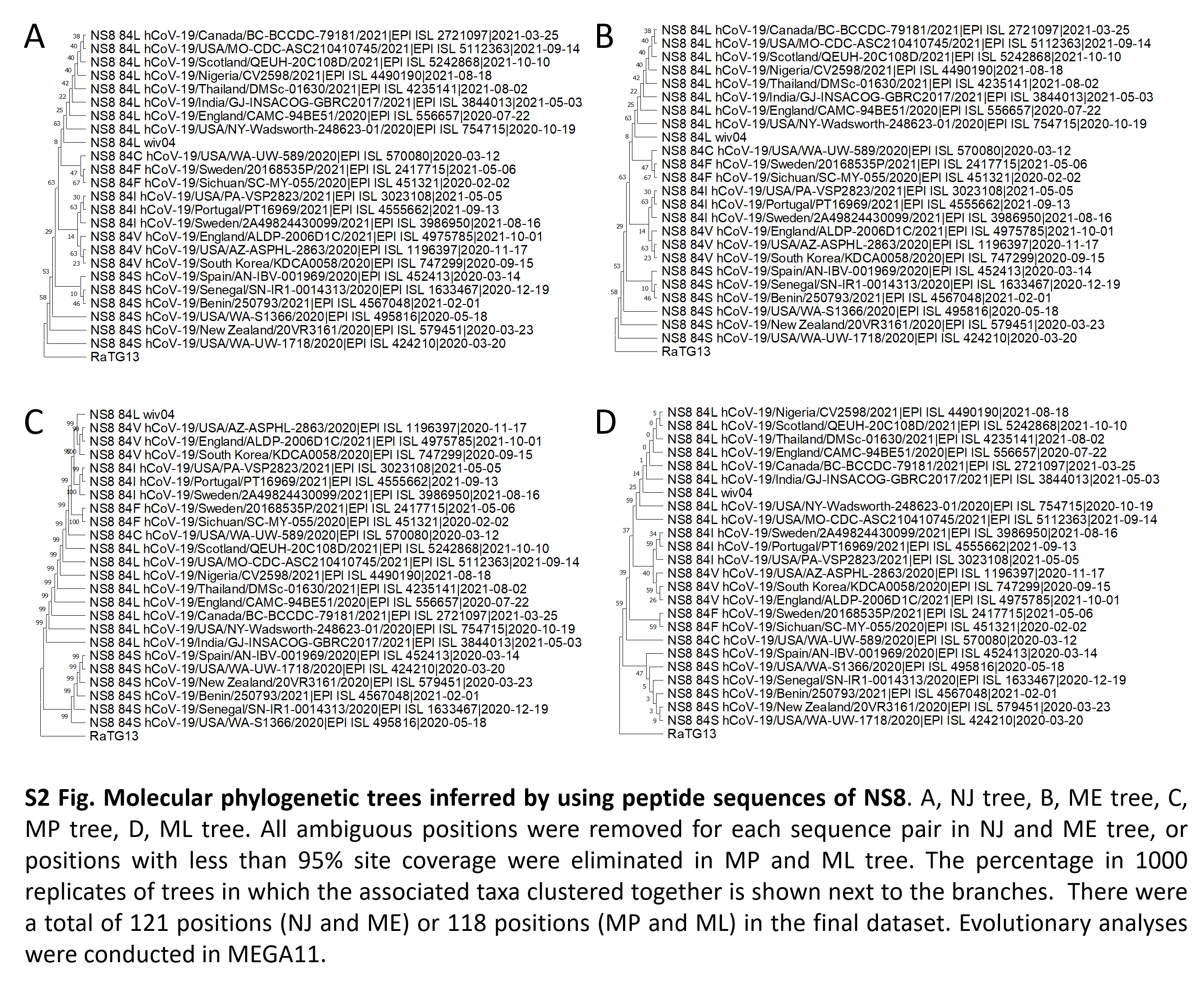

Supplement: S2 Fig — A, NJ tree, B, ME tree, C, MP tree, D, ML tree. All ambiguous positions were removed for each sequence pair in NJ and ME tree, or positions with less than 95% site coverage were eliminated in MP and ML tree. The percentage in 1000 replicates of trees in which the associated taxa clustered together is shown next to the branches. There are a total of 121 positions (NJ and ME) or 118 positions (MP and ML) in the final dataset. Evolutionary analyses were conducted in MEGA11 [36]. (TIF) [file pone.0279221.s002.tif]

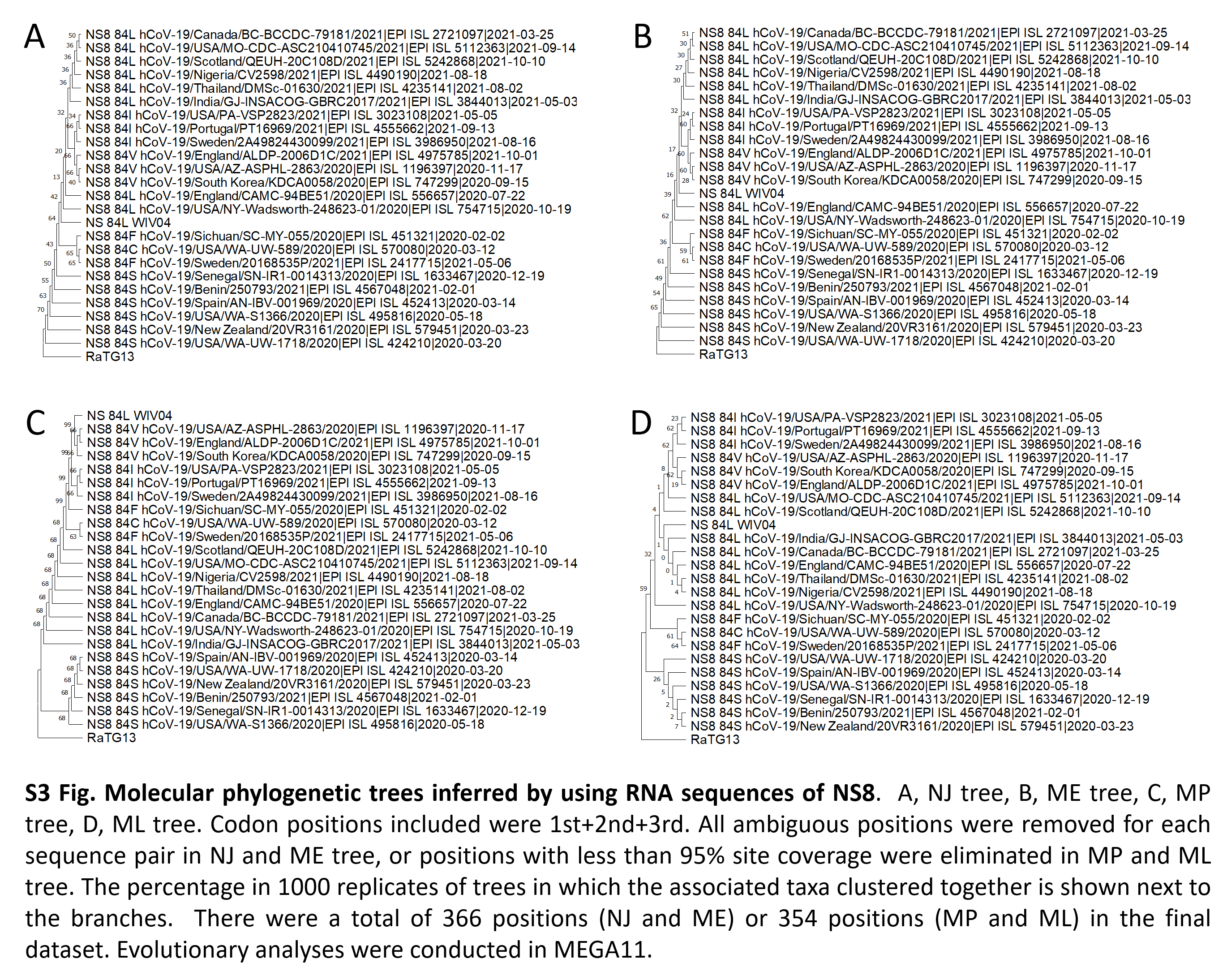

Supplement: S3 Fig — A, NJ tree, B, ME tree, C, MP tree, D, ML tree. Codon positions included were 1st+2nd+3rd. All ambiguous positions were removed for each sequence pair in NJ and ME tree, or positions with less than 95% site coverage were eliminated in MP and ML tree. The percentage in 1000 replicates of trees in which the associated taxa clustered together is shown next to the branches. There are a total of 366 positions (NJ and ME) or 354 positions (MP and ML) in the final dataset. (TIF) [file pone.0279221.s003.tif]

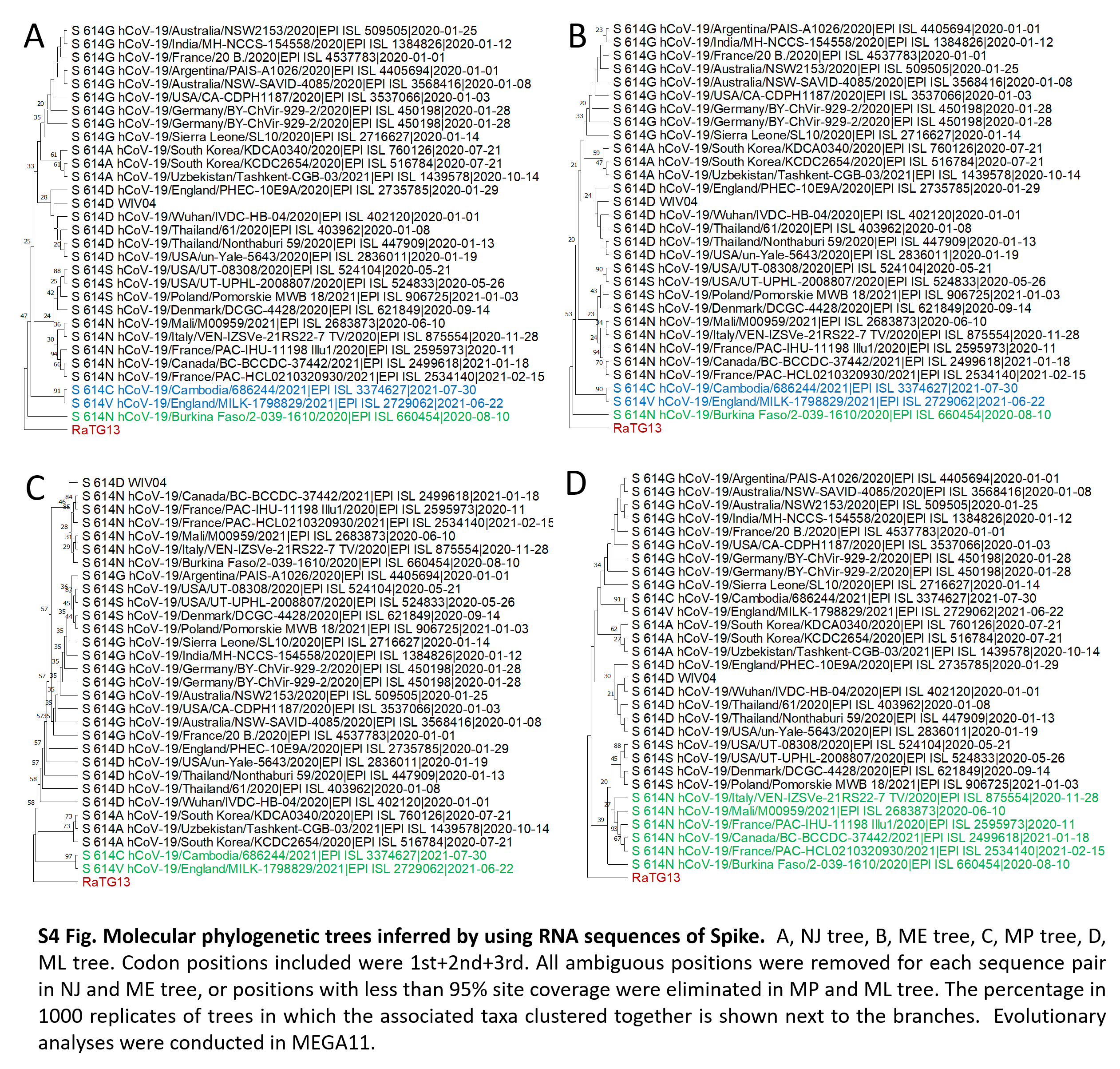

Supplement: S4 Fig — A, NJ tree, B, ME tree, C, MP tree, D, ML tree. Codon positions included were 1st+2nd+3rd. All ambiguous positions were removed for each sequence pair in NJ and ME tree, or positions with less than 95% site coverage were eliminated in MP and ML tree. The percentage in 1000 replicates of trees in which the associated taxa clustered together is shown next to the branches. (TIF) [file pone.0279221.s004.tif]

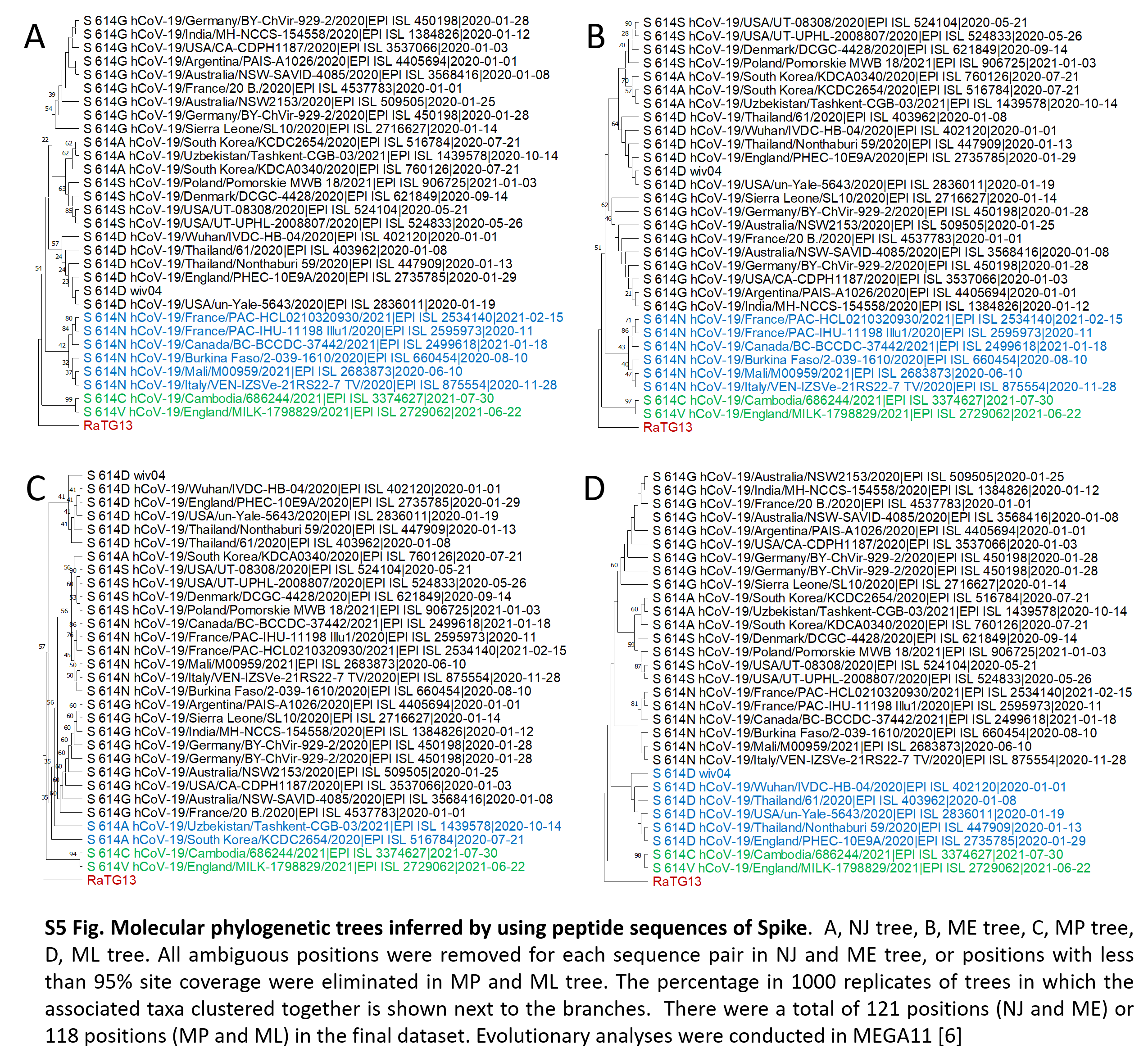

Supplement: S5 Fig — A, NJ tree, B, ME tree, C, MP tree, D, ML tree. All ambiguous positions were removed for each sequence pair in NJ and ME tree, or positions with less than 95% site coverage were eliminated in MP and ML tree. The percentage in 1000 replicates of trees in which the associated taxa clustered together is shown next to the branches. There are a total of 121 positions (NJ and ME) or 118 positions (MP and ML) in the final dataset. (TIF) [file pone.0279221.s005.tif]

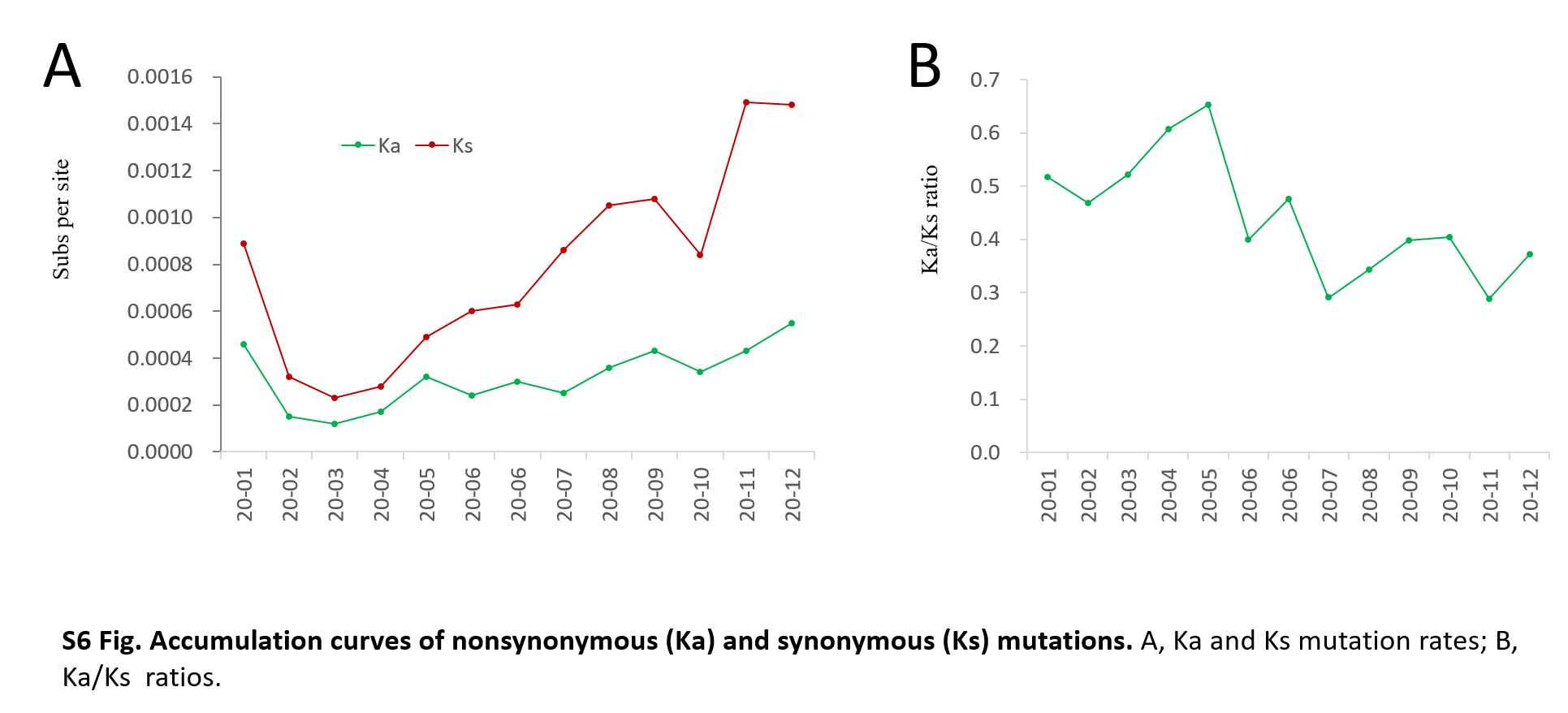

Supplement: S6 Fig — A, Ka and Ks mutation rates; B, Ka/Ks ratios. (TIF) [file pone.0279221.s006.tif]

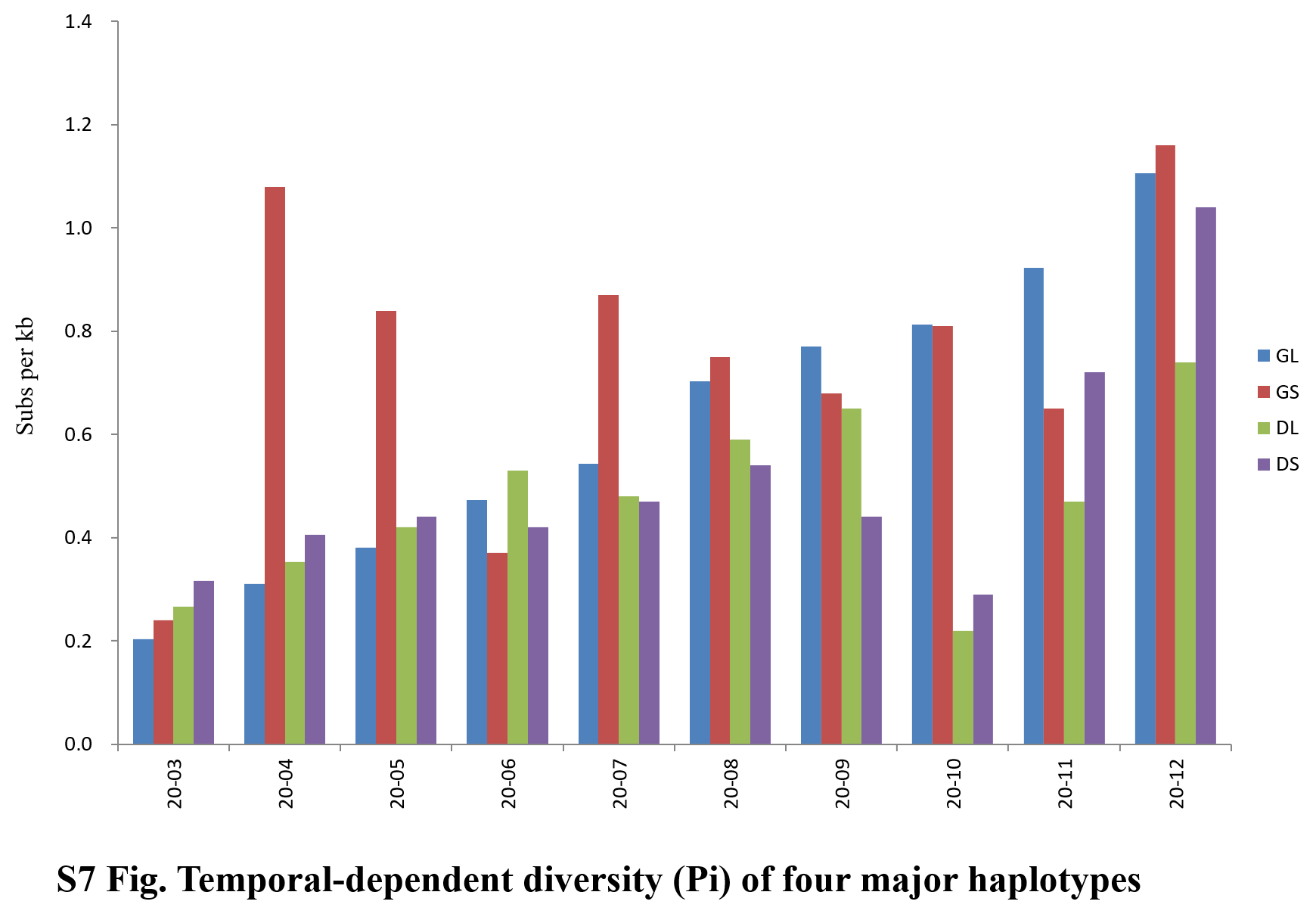

Supplement: S7 Fig — (TIF) [file pone.0279221.s007.tif]

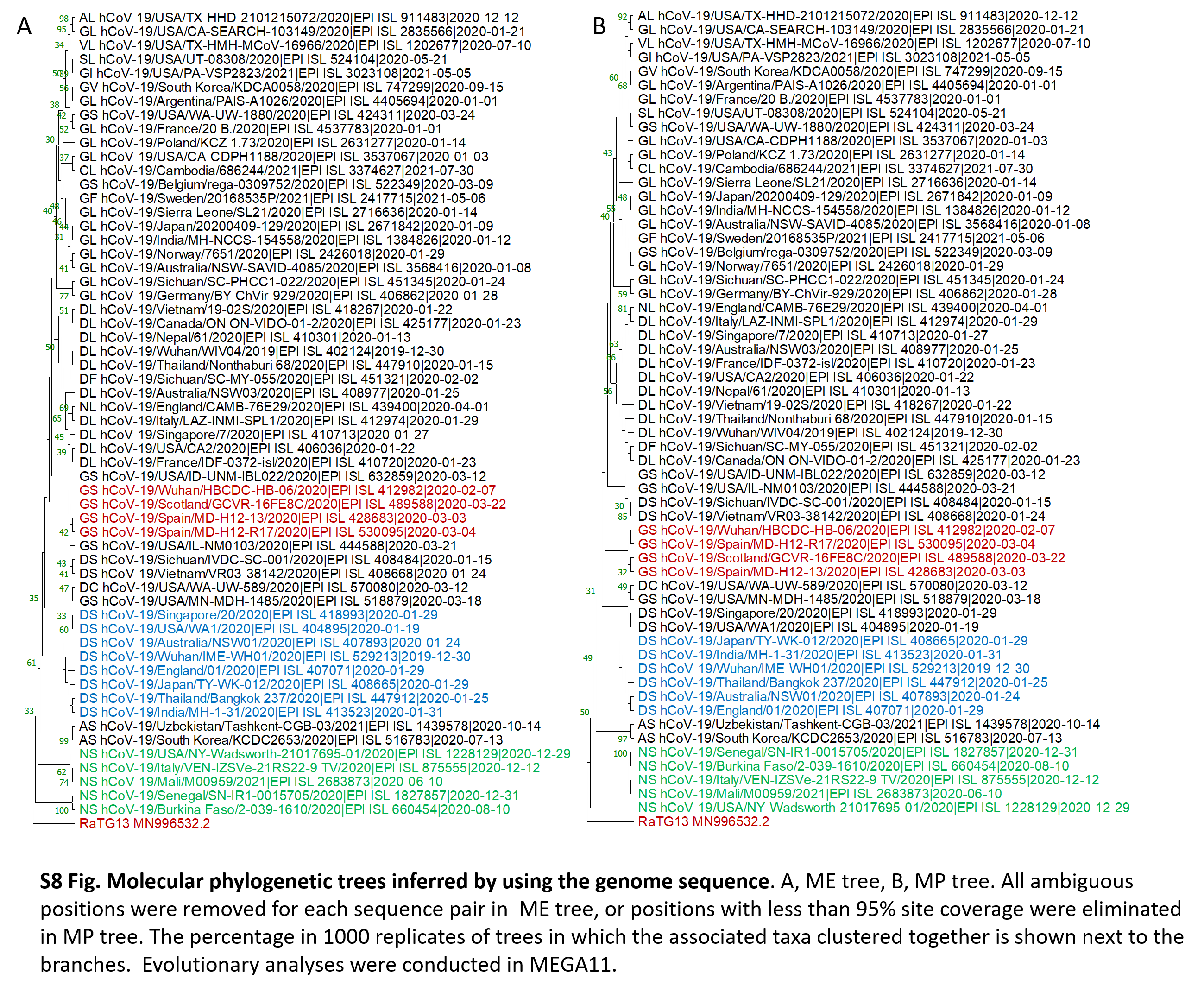

Supplement: S8 Fig — A, ME tree, B, MP tree. All ambiguous positions were removed for each sequence pair in ME tree, or positions with less than 95% site coverage were eliminated in MP tree. The percentage in 1000 replicates of trees in which the associated taxa clustered together is shown next to the branches. (TIF) [file pone.0279221.s008.tif]
