# Supplementary material for: Genome characterization based on the Spike-614 and NS8-84 loci of SARS-CoV-2 reveals two major possible onsets of the COVID-19 pandemic
Source: PLoS One. 2023 Jun 15;18(6):e0279221. doi: 10.1371/journal.pone.0279221 (PMC10270620; doi:10.1371/journal.pone.0279221)
Supplement: S1 File — (PDF) [file pone.0279221.s013.pdf]

S1 File. Metadata and genomes used in this study

Data Availability

GISAID Identifier:	EPI_SET_230423ot
doi:	10.55876/gis8.230423ot

All genome sequences and associated metadata in this dataset are published in GISAID's EpiCoV database. To view the contributors of each individual sequence with details such as accession number, Virus name, Collection date, Originating Lab and Submitting Lab and the list of Authors, visit 10.55876/gis8.230423ot

Data Snapshot

- EPI_SET_230423ot is composed of 3,244,841 individual genome sequences.
- The collection dates range from 2019-12-24 to 2021-10-16;
- Data were collected in 187 countries and territories;
- All sequences in this dataset are compared relative to hCoV-19/Wuhan/WIV04/2019 (WIV04), the official reference sequence employed by GISAID (EPI_ISL_402124). Learn more at https://gisaid.org/WIV04.
