# Supplementary material for: Genome characterization based on the Spike-614 and NS8-84 loci of SARS-CoV-2 reveals two major possible onsets of the COVID-19 pandemic
Source: PLoS One. 2023 Jun 15;18(6):e0279221. doi: 10.1371/journal.pone.0279221 (PMC10270620; doi:10.1371/journal.pone.0279221)
Supplement: Attachment — Submitted filename: PONE-D-22-33104 reviewer1 comment.docx [file pone.0279221.s014.docx]

Dear PLOS ONE Editor,

Find below my comments on the article titled: Genome charaterization based on the Spike-614 and NS8-84 loci of SARS-CoV-2 reveals two major onsets of the COVID-19 pandemic.

The article requires minor corrections of typos and other errors.

Thank you.

**Minor Correction:**

Title: Consider the use of the word ‘…two major **possible** onsets of the COVID-19 pandemic’.

Page 1, line 1: The word **characterization** is wrongly spelled as ‘charaterization’.

Page 3, line 7: The date (April 2019.) mentioned therein is erroneous.

Page 4, line 15: The word **uncovered** seems unintended in the statement ‘the origin of the virus remains uncovered’.

Page 6, line 16: The words ‘sites to be’ and ‘early’ could be expunged to avoid repetitions in the sentence.

Page 7, Table 1: The last entry designated as ‘unknown’ contain incomplete information. Kindly provide the complete information or expunge the entire cells. It may be more meaningful to fill unknowns into the cells rather than leave them blank.

Page 7, line 7: The word ‘Genome’ may not be necessary, hence the subheading may be written as ‘Mutation accumulation….’.

Page 7, Line 17: Kindly confirm that the date quoted ‘14 October 2019’ was correctly inferred from Fig 2E.

Page 7, Line 18: Kindly confirm that the date quoted ‘28 September 2019’ was correctly inferred from Fig 2F.

Page 8, line 5: The word ‘consistent’ could be expunged or corrected to the adverbial form ‘consistently’.

Page 8, lines 5 and 6: Kindly recast the phrase ‘which was supported by that the bat SARS-CoV related viruses all have NS8_84S’.

Page 8, line 16: Mutation S_614V was written twice.

Page 9, Table 2: The last entry designated as ‘unknown’ contain incomplete information. Kindly provide the complete information or expunge the entire cells. It may be more meaningful to fill unknowns into the cells rather than leave them blank.

Page 13, line 19: …went distinct. Do you mean to write extinct?

Page 13, line 27: Despite **of** tremendous efforts… Remove the ‘of’.

Page 13, line 29: ‘SARS-CoV-1 that caused a pandemic in China in 2003’. Ensure to correctly capture the burden and spread of the said event. A pandemic may begin in China and spread to other countries, but if the statement suggests that it affects only China, then it is an epidemic.

Figure 1: Figure 1 was not well labeled; hence it was difficult to identify which of the figures is ‘A’, ‘B’ etc.

Page 22, line 14: Figure Legend; there should be a conjunction between genomes **and** confirmed.
